# Volumetric breast density affects performance of digital screening mammography

**DOI:** 10.1007/s10549-016-4090-7

**Published:** 2016-12-23

**Authors:** Johanna O. P. Wanders, Katharina Holland, Wouter B. Veldhuis, Ritse M. Mann, Ruud M. Pijnappel, Petra H. M. Peeters, Carla H. van Gils, Nico Karssemeijer

**Affiliations:** 1 Julius Center for Health Sciences and Primary Care, University Medical Center Utrecht, P.O. Box 85500, 3508 GA Utrecht, The Netherlands; 2 Department of Radiology and Nuclear Medicine, Radboud University Medical Center, Geert Grooteplein 10, 6525 GA Nijmegen, The Netherlands; 3 Department of Radiology, University Medical Center Utrecht, P.O. Box 85500, 3508 GA Utrecht, The Netherlands; 4 Dutch Reference Centre for Screening, Postbus 6873, 6503 GJ Nijmegen, The Netherlands; 5 MRC-PHE Centre for Environment and Health, Department of Epidemiology and Biostatistics, School of Public Health, Imperial College, London, St. Mary’s Campus, Norfolk Place W2 1PG, London, UK

**Keywords:** Mammographic density, Breast cancer, Cancer screening, Mammography, Breast

## Abstract

**Purpose:**

To determine to what extent automatically measured volumetric mammographic density influences screening performance when using digital mammography (DM).

**Methods:**

We collected a consecutive series of 111,898 DM examinations (2003–2011) from one screening unit of the Dutch biennial screening program (age 50–75 years). Volumetric mammographic density was automatically assessed using Volpara. We determined screening performance measures for four density categories comparable to the American College of Radiology (ACR) breast density categories.

**Results:**

Of all the examinations, 21.6% were categorized as density category 1 (‘almost entirely fatty’) and 41.5, 28.9, and 8.0% as category 2–4 (‘extremely dense’), respectively. We identified 667 screen-detected and 234 interval cancers. Interval cancer rates were 0.7, 1.9, 2.9, and 4.4‰ and false positive rates were 11.2, 15.1, 18.2, and 23.8‰ for categories 1–4, respectively (both *p*-trend < 0.001). The screening sensitivity, calculated as the proportion of screen-detected among the total of screen-detected and interval tumors, was lower in higher density categories: 85.7, 77.6, 69.5, and 61.0% for categories 1–4, respectively (*p*-trend < 0.001).

**Conclusions:**

Volumetric mammographic density, automatically measured on digital mammograms, impacts screening performance measures along the same patterns as established with ACR breast density categories. Since measuring breast density fully automatically has much higher reproducibility than visual assessment, this automatic method could help with implementing density-based supplemental screening.

## Introduction

Breast density increases breast cancer risk [1, 2]. In addition, sensitivity of screening mammography is lower for women with dense breasts, caused by the masking effect of dense (fibroglandular) breast tissue [3, 4]. This has led to breast density legislation in 28 states of the United States of America (USA) until now, and has fueled ongoing discussions on the need for supplemental screening for women with dense breasts worldwide [5].

One hoped that screening performance in women with dense breasts would improve when film-screen mammography (FSM) was replaced by digital mammography (DM). Unfortunately, screening sensitivity was still worse in women with dense compared to nondense breasts when DM was used [6–8]. Most large studies looking into the effect of breast density on screening performance used the breast imaging reporting and data system (BI-RADS) for breast density assessment, which is assessed by radiologists. However, this method has a moderate inter-observer agreement [9–12].

With the advent of digital mammography, several fully automatic volumetric density assessment methods have been developed. Volpara is one of these methods, and has shown correlation with BI-RADS density categories and MRI breast density measurements [13–16].

The effect of automatically measured volumetric breast density on screening sensitivity has only been studied once [17]. However, information about the effect of automatically measured volumetric breast density on other screening performance measures like recall rates, false positive rates, and positive predictive values (PPV) was not given in this study. Therefore, the aim of this study was to examine to what extent automatically measured volumetric mammographic density affected screening sensitivity and other screening performance measures in a large Dutch population-based screening program cohort containing a consecutive series of digital screening mammograms and complete information about interval cancers.

## Materials and methods

### Study population

Data were acquired from a breast cancer screening unit (Preventicon screening unit 19, Utrecht, the Netherlands) of the Foundation of Population Screening Mid-West, one of the five screening regions of the Dutch breast cancer screening program. Women participating in this biennial screening program are aged 50–75. The program involves mammography only, and all mammograms are read by two certified screening radiologists. In the Dutch screening program, previous screening mammograms are most of the time available for comparison in case of subsequent screens.

In 2003, DM was introduced at the Preventicon screening unit [18–20]. Analog mammography systems were gradually replaced by digital ones. In July 2007, almost all mammograms at this screening unit were digital [19].

By participating in the Dutch screening program, women consent to their data being used for evaluation and improvement of the screening, unless they have indicated otherwise.

### Data collection

We prospectively collected all unprocessed DM examinations that were taken at the Preventicon screening unit between 2003 and 2011, with exception of a 4-month period in 2009 when only processed data were archived. All mammograms were acquired using Lorad Selenia DM systems (Hologic, Danbury, Conn.). The first screening examination of a woman in the screening program always included the two standard views, craniocaudal (CC) and mediolateral oblique (MLO). At subsequent screening examinations, MLO was the routinely acquired view and CC was acquired in 57% of the cases by indication (e.g., high breast density, visible abnormality) during the study period. Recall and breast cancer detection information was obtained from the screening registration system. Interval cancers were identified through linkage with the Netherlands Cancer Registry.

Examinations were excluded, when information about recall or final outcome was missing. In addition, examinations for which breast density could not be determined, and interval cancers diagnosed more than 24 months after the last screening mammogram were excluded for analysis.

Tumor information such as maximum diameter, nodal status, and ICD-O codes were obtained from the screening registration system. Nodes were classified negative when the sentinel lymph node, or the dissection specimen in case no sentinel lymph node procedure was performed, contained no or only isolated tumor cells. Nodes were considered positive if they contained micrometastases (0.2–2 mm) or metastases larger than 2 mm.

### Volumetric mammographic density assessment

Percentage dense volume (PDV) was automatically assessed from unprocessed mammograms of the left and right breasts, and MLO and CC views using the commercially available Volpara Density software (version 1.5.0, Volpara Solutions, Wellington, New Zealand) [21].

The average PDV per screening examination was determined using the available views of both breasts. Volpara density grades (VDGs) were constructed based on this average PDV (VDG1: 0% ≤ VBD < 4.5%, VDG2: 4.5% ≤ VBD < 7.5%, VDG3: 7.5% ≤ VBD < 15.5%, VDG4: VBD ≥ 15.5%). The VDGs are designed to mimic the American College of Radiology (ACR) BI-RADS breast density categories (4th edition).

### Statistical analysis

Examinations were grouped according to VDGs. Within these groups, we determined the following screening performance measures with accompanying 95% confidence intervals (CI) using generalized estimating equations (GEE) to account for correlation between examinations of the same woman using the ‘independence’ correlation structure: recall rate, false positive rate, screen-detected breast cancer rate, interval breast cancer rate, total breast cancer rate (all rates are per 1000 screening examinations), sensitivity and specificity of the screening, and positive predictive value (PPV). For the screening sensitivity, we calculated Wilson’s 95% confidence intervals (see Table 1 for screening performance definitions). For comparison with American screening programs, we also determined interval cancer rates for the first year after a negative screening mammogram, since the screening interval in the USA is normally 1 year.

**Table 1:** Definitions of screening performance measures

FN (Interval breast cancer)	Breast cancers diagnosed within 24 months after a screening examination that did not lead to recall (negative mammogram), and before the next scheduled screening examination
TP (Screen-detected breast cancer)	Breast cancers diagnosed after a recalled screening examination (positive mammogram)
FP	Screening examinations that led to a recall (positive mammogram), but not to a breast cancer diagnosis within 24 months after the examination, or before the next scheduled screening examination
TN	Screening examinations that did not lead to recall (negative mammogram) and no breast cancer was diagnosed within 24 months after the examination, or before the next scheduled screening examination
Sensitivity of screening	The number of screen-detected breast cancers divided by the total number of screen-detected plus interval breast cancers ((TP/(TP + FN))
Specificity of screening	Number of screening examinations that did not lead to recall (negative mammogram) and no breast cancer diagnosis within 24 months, or before the next scheduled screening examination divided by the total number of examinations without breast cancer diagnosis within 24 months, or before the next scheduled screening examination ((TN/(TN + FP))
PPV	The number of screen-detected breast cancers divided by the total number of examinations that led to recall ((TP/(TP + FP))

*FN* false negative, *TP* true positive, *FP* false positive, *TN* true negative, *PPV* positive predictive value

 We performed several sensitivity analyses: (1) taking only invasive tumors into account (i.e., excluding the examinations leading to a true positive or false negative diagnosis of in situ carcinoma); (2) taking only subsequent screening rounds into account, since performance measures are expected to be different between first and subsequent rounds (in case of subsequent rounds, the prior mammogram could be analog or digital); (3) using VDGs based on the mean PDV of only the MLO views instead of using all available views.

We tested for linear trends across the four density categories for screening performance measures, the percentage of in situ cancers, and positive lymph nodes with a Chi square linear trend test. In addition, we examined whether tumors diagnosed in dense breasts were larger than in nondense breasts, using the Jonckheere-Terpstra test, as we expected tumor size not to be normally distributed. All statistical tests were two-sided. Statistical analyses were performed in IBM SPSS statistics, version 21 and in R, version 3.2.2 using the “geese” function from the “geepack” package.

## Results

In total, 113,956 screening examinations were available. We excluded 50 examinations of which the screening outcome was unknown, 47 interval cancers which were diagnosed more than 24 months after the last screening examination, and 1961 examinations for which VDG could not be assessed. This resulted in 111,898 examinations belonging to 53,239 women with a median age of 58 years (IQR: 53–64 years). Among the examinations, 21.6% were categorized as density category 1 (‘almost entirely fatty’), and 41.5, 28.9, and 8.0% as category 2–4 (‘extremely dense’), respectively (Table 2). In total, 667 screen-detected breast cancers were identified based on a mammogram taken before January 1, 2012, and 234 interval cancers were identified within 24 months after a mammogram taken before January 1, 2012, of which 79.5 and 97.9%, respectively, were invasive breast cancers (Tables 2, 4).

**Table 2:** Number of mammography examinations in total and within Volpara Density Grade (VDG) categories (based on the available views)

	Total	VDG 1	VDG 2	VDG 3	VDG 4
Total					
Screening examinations [*N* (%)]	111,898 (100%)	24,210 (21.6%)	46,426 (41.5%)	32,330 (28.9%)	8932 (8.0%)
Screen-detected cancers (*N*)	667	96	298	212	61
Interval cancers (*N*)	234	16	86	93	39
False positives (*N*)	1774	271	700	590	213
True negatives (*N*)	109,223	23,827	45,342	31,435	8619
Only invasive tumors taken into account					
Screening examinations [*N* (%)]	111,754 (100%)	24,188 (21.6%)	46,375 (41.5%)	32,279 (28.9%)	8912 (8.0%)
Screen-detected cancers (*N*)	529	75	250	163	41
Interval cancers (*N*)	228	15	83	91	39
False positives (*N*)	1774	271	700	590	213
True negatives (*N*)	109,223	23,827	45,342	31,435	8619
Only subsequent screening rounds					
Screening examinations [*N* (%)]	94,665 (100%)	22,146 (23.4%)	40,664 (43.0%)	25,777 (27.2%)	6078 (6.4%)
Screen-detected cancers (*N*)	521	86	249	152	34
Interval cancers (*N*)	203	16	81	80	26
False positives (*N*)	1170	214	491	366	99
True negatives (*N*)	92,771	21,830	39,843	25,179	5919

### Screening performance across volumetric density categories

Table 3 shows that total and interval breast cancer rates, recall rates, and false positive rates were higher in higher breast density categories compared to lower density categories, all with a significant linear trend (*p*-trend < 0.001). Screen-detected breast cancer rates were found to be lowest in the lowest breast density category (4.0 per 1000 examinations (‰)) and more comparable across the three highest breast density categories: 6.4, 6.6, and 6.8‰, respectively (*p*-trend < 0.001). The screening sensitivity was significantly lower (*p*-trend < 0.001) in higher breast density categories: 85.7, 77.6, 69.5, and 61.0% in VDG categories 1–4, respectively. No significant linear trend was found for PPV (*p*-trend = 0.12) (Table 3).

**Table 3:** Screening performance measures in total and within volpara density grade (VDG) categories (based on the available views)

	Screening performance measures (95% CI) for total population and within VDG breast density categories					*p* trend
	Total	VDG 1	VDG 2	VDG 3	VDG 4	
Total						
Recall/1000	21.8 (20.9; 22.7)	15.2 (13.7; 16.8)	21.7 (20.2; 22.9)	24.8 (23.1; 26.6)	30.7 (27.2; 34.5)	<0.001
FP/1000	15.9 (15.1; 16.6)	11.2 (9.9; 12.6)	15.1 (14.0; 16.2)	18.2 (16.8; 19.8)	23.8 (20.8; 27.3)	<0.001
Screen-detected cancer/1000	6.0 (5.5; 6.4)	4.0 (3.2; 4.8)	6.4 (5.7; 7.2)	6.6 (5.7; 7.5)	6.8 (5.3; 8.8)	<0.001
Interval cancer/1000	2.1 (1.9; 2.4)	0.7 (0.4; 1.1)	1.9 (1.5; 2.3)	2.9 (2.3; 3.5)	4.4 (3.2; 6.0)	<0.001
BC/1000	8.1 (7.6; 8.7)	4.6 (3.8; 5.6)	8.3 (7.5; 9.1)	9.4 (8.4; 10.5)	11.2 (9.2; 13.6)	<0.001
Sensitivity of screening (%)	74.0 (71.1; 76.7)	85.7 (78.1; 91.0)	77.6 (73.2; 81.5)	69.5 (64.1; 74.4)	61.0 (51.2; 70.0)	<0.001
Specificity (%)	98.4 (98.3; 98.5)	98.9 (98.7; 99.0)	98.5 (98.4; 98.6)	98.2 (98.0; 98.3)	97.6 (97.2; 97.9)	<0.001
PPV (%)	27.3 (25.6; 29.1)	26.2 (21.9; 30.9)	29.9 (27.1; 32.8)	26.4 (23.5; 29.6)	22.3 (17.7; 27.6)	0.12
Only invasive tumors taken into account						
Recall/1000	20.6 (19.8; 21.4)	14.3 (12.9; 15.9)	20.5 (19.2; 21.8)	23.3 (21.7; 25.1)	28.5 (25.2; 32.3)	<0.001
FP/1000	15.9 (15.1; 16.6)	11.2 (9.9; 12.6)	15.1 (14.0; 16.3)	18.3 (16.9; 19.8)	23.9 (20.9; 27.4)	<0.001
Screen-detected cancer/1000	4.7 (4.3; 5.1)	3.1 (2.5; 3.9)	5.4 (4.8; 6.1)	5.0 (4.3; 5.9)	4.6 (3.4; 6.2)	0.02
Interval cancer/1000	2.1 (1.9; 2.4)	0.6 (0.4; 1.0)	1.8 (1.4; 2.2)	2.8 (2.3; 3.5)	4.4 (3.2;6.0)	<0.001
BC/1000	6.9 (6.4; 7.3)	3.7 (3.0; 4.6)	7.2 (6.5; 8.0)	7.9 (7.0; 8.9)	9.0 (7.2; 11.1)	<0.001
Sensitivity of screening (%)	69.1 (66.5; 73.0)	83.3 (74.3; 89.6)	74.4 (70.2; 79.4)	62.9 (58.1; 69.8)	50.6 (40.5; 61.9)	<0.001
Specificity (%)	98.4 (98.3; 98.5)	98.9 (98.7; 99.0)	98.5 (98.4; 98.6)	98.2 (98.0; 98.3)	97.6 (97.2; 97.9)	<0.001
PPV (%)	23.0 (21.3; 24.7)	21.7 (17.6; 26.3)	26.3 (23.6; 29.2)	21.6 (18.9; 24.7)	16.1 (12.1; 21.2)	0.02
Only subsequent screening rounds taken into account						
Recall/1000	17.9 (17.0; 18.7)	13.5 (12.1; 15.2)	18.2 (16.9; 19.5)	20.1 (18.4; 21.9)	21.9 (18.5; 25.9)	<0.001
FP/1000	12.4 (11.7; 13.1)	9.7 (8.4; 11.0)	12.1 (11.1; 13.2)	14.2 (12.8; 15.7)	16.3 (13.3; 19.9)	<0.001
Screen-detected cancer/1000	5.5 (5.0; 6.0)	3.9 (3.1; 4.8)	6.1 (5.4; 6.9)	5.9 (5.0; 6.9)	5.6 (4.0; 7.8)	0.02
Interval cancer/1000	2.2 (1.9; 2.5)	0.7 (0.4; 1.2)	2.0 (1.6; 2.5)	3.1 (2.5; 3.9)	4.3 (2.9; 6.3)	<0.001
BC/1000	7.7 (7.2; 8.3)	4.6 (3.8; 5.6)	8.1 (7.3; 9.0)	9.0 (7.9; 10.2)	9.9 (7.7; 12.7)	<0.001
Sensitivity of screening (%)	71.3 (68.6; 75.1)	84.3 (76.0; 90.1)	74.8 (70.1; 79.8)	64.4 (59.2; 71.3)	56.7 (44.1; 68.4)	<0.001
Specificity (%)	98.8 (98.7; 98.8)	99.0 (98.9; 99.2)	98.8 (98.7; 98.9)	98.6 (98.4; 98.7)	98.4 (98.0; 98.7)	<0.001
PPV (%)	30.8 (28.6; 33.0)	28.7 (23.8; 34.0)	33.6 (30.3; 37.1)	29.3 (25.6; 33.4)	25.6 (18.8; 33.7)	0.35

*FP* false positive examinations, *BC* breast cancers, *PPV* positive predictive value. BC/1000 = (Screen-detected cancers/1000) + (Interval cancers/1000), Sensitivity of screening = screen-detected cancers/(screen-detected cancers + interval cancers), Specificity = true negative examinations/(true negative examinations + false positive examinations), PPV = screen-detected cancers/(screen-detected cancers + false positive examinations)

Overall trends for interval cancer rates, recall rates and false positive rates, screening sensitivity and specificity were similar when either invasive cancers alone or both invasive cancer and in situ cancers were taken into account. However, when restricting the analyses to invasive cancers only, the screening sensitivity in VDG4 decreased most notably compared to the screening sensitivity when both in situ and invasive breast cancers were taken into account. When only subsequent screening rounds were taken into account, the overall trends were again similar to the analyses based on both first and subsequent screening examinations (Table 3). The results of the sensitivity analysis, where PDV was based on MLO views only, did not differ from those based on all available views (data not shown).

In VDG category 1, 25% of the interval breast cancers were diagnosed in the first year after screening examination; in VDG categories 2 and 3, this was 41% and in VDG category 4 67%. This resulted in interval cancer rates in the first year after a screening examination of 0.2, 0.8, 1.2, and 2.9‰ (*p*-trend < 0.001) in VDG categories 1, 2, 3, and 4, respectively.

### Tumor characteristics across volumetric density categories

Of all tumors, 74.0% were screen-detected and 26.0% were interval cancers. 15.7% of all tumors were in situ and 84.3% were invasive tumors. 89.4% of the in situ tumors showed microcalcifications on the last screening mammogram. For screen-detected tumors, the highest proportion of in situ tumors was found in the highest density category (in VDG4, 32.8% of the screen-detected tumors were in situ tumors) and the lowest proportion in density category 2 (in VDG2, 15.8% of the screen-detected tumors were in situ tumors). A significant linear trend was observed for the proportion of invasive tumors over breast density categories among screen-detected tumors (*p*-trend = 0.03).

About 80% of the screen-detected and slightly over 50% of the interval invasive breast cancers were smaller than 20 mm (pT1 status) at diagnosis. No linear trend was found for screen-detected tumor size across the four density categories (*p*-trend_SD_ = 0.10) (Table 4). Lymph nodes were positive in 29.3% of the screen-detected cancers and 36.8% of the interval cancers. For lymph node status, no linear trend was found across the four breast density categories for screen-detected breast cancers (*p*-trend_SD_ = 0.08) (Table 4).

**Table 4:** Tumor characteristics in total and within Volpara Density Grade (VDG) categories (based on the available views)

		Total	VDG 1	VDG 2	VDG 3	VDG 4	*p* trend
Proportion invasive tumors^a^							
Total (*N* = 898)	Invasive [*N* (%)]	757 (84.3%)	90 (80.4%)	333 (87.2%)	254 (83.6%)	80 (80.0%)	0.49
Screen-detected cancer (*N* = 665)	Invasive [*N* (%)]	529 (79.5%)	75 (78.1%)	250 (84.2%)	163 (77.3%)	41 (67.2%)	0.03
Interval cancer (*N* = 233)	Invasive [*N* (%)]	228 (97.9%)	15 (93.8%)	83 (97.6%)	91 (97.8%)	39 (100.0%)	0.20
pT (only invasive tumors)^b^							
Total (*N* = 700)	T1 [*N* (%)]	503 (71.9%)	70 (81.4%)	231 (73.6%)	153 (66.8%)	49 (69.0%)	
	T2 [*N* (%)]	171 (24.4%)	15 (17.4%)	74 (23.6%)	65 (28.4%)	17 (23.9%)	0.02^c^
	T3 & T4 [*N* (%)]	26 (3.7%)	1 (91.2%)	9 (2.9%)	11 (4.8%)	5 (7.0%)	
Screen-detected cancer (*N* = 511)	T1 [*N* (%)]	404 (79.1%)	63 (85.1%)	195 (79.6%)	116 (75.8%)	30 (76.9%)	
	T2 [*N* (%)]	97 (19.0%)	11 (14.9%)	46 (18.8%)	33 (21.6%)	7 (17.9%)	0.14^c^
	T3 & T4 [*N* (%)]	10 (2.0%)	0 (0.0%)	4 (1.6%)	4 (2.6%)	2 (5.1%)	
Interval cancer (*N* = 189)	T1 [*N* (%)]	99 (52.4%)	7 (58.3%)	36 (52.2%)	37 (48.7%)	19 (59.4%)	
	T2 [*N* (%)]	74 (39.2%)	4 (33.3%)	28 (40.6%)	32 (42.1%)	10 (31.3%)	0.87^c^
	T3 and T4 [*N* (%)]	16 (8.5%)	1 (8.3%)	5 (7.2%)	7 (9.2%)	3 (9.4%)	
Lymph node status (only invasive tumors)^d^							
Total (*N* = 741)	Positive [*N* (%)]	234 (31.6%)	18 (20.2%)	105 (32.3%)	87 (35.2%)	24 (30.0%)	0.12
Screen-detected cancer (*N* = 518)	Positive [*N* (%)]	152 (29.3%)	13 (17.6%)	75 (30.7%)	51 (32.1%)	13 (31.7%)	0.08
Interval cancer (*N* = 223)	Positive [*N* (%)]	82 (36.8%)	5 (33.3%)	30 (37.0%)	36 (40.9%)	11 (28.2%)	0.68
Tumor diameter (only invasive tumors)^e^							
Total (*N* = 691)	Median (mm) (IQR)	15 (10; 22)	12 (8; 18)	15 (10; 21)	17 (11; 25)	14 (10; 22)	0.01
Screen-detected cancer (*N* = 500)	Median (mm) (IQR)	13 (9; 19)	11 (8; 17)	13 (10; 19)	14 (10; 20)	12 (8; 19)	0.10
Interval cancer (*N* = 191)	Median (mm) (IQR)	20 (14; 30)	20 (13; 33)	19 (16; 30)	21 (16; 31)	16 (12; 25)	0.34

^a^Information on invasiveness is missing for 3 tumors (2 screen-detected and 1 interval tumors)

^b^Information on pT status is missing for 57 tumors (18 screen-detected and 39 interval tumors)

^c^ *p*-trend determined for T1 versus T2, T3, and T4

^d^Information on lymph node status is missing for 16 tumors (11 screen-detected and 5 interval tumors)

^e^Information on tumor diameter is missing for 66 tumors (29 screen-detected and 37 interval tumors)

## Discussion

We found that the sensitivity of a DM screening program was significantly lower in women with high volumetric breast density than in women with low volumetric breast density (61.0 and 85.7%, respectively, (*p*-trend < 0.001)). This is despite the higher recall rates in women with high compared to low breast density (30.7 and 15.2‰, respectively) (*p*-trend < 0.001).

A study of Destounis et al., which was recently published, also studied the screening sensitivity in four automatically determined volumetric breast density categories. They found screening sensitivities of 95, 89, 83, and 65% in density categories 1–4, respectively. Additionally, they determined the mammographic screening sensitivity across the visual BI-RADS categories and found sensitivities of 82% in the lowest and 66% in the highest breast density category [17].

Four other studies where breast density was visually assessed on digital screening mammograms, also found a negative influence of breast density on screening sensitivity [6–8, 22] a fifth study did not find this result [23]. A Canadian study showed a lower screening sensitivity for women with 75% or higher breast density (74.2% (95% CI 67.2–80.4)) compared to women with less than 75% breast density (80.2% (95% CI 78.4–81.9)) when using direct radiography (DR) in a biennial screening program, where women who are considered to be at increased risk were screened annually [8]. In the American Digital Mammographic Imaging Screening Trial (DMIST), the screening sensitivity was determined for women with dense and nondense breasts for several subgroups. Sensitivity seemed higher for all nondense compared to dense subgroup comparisons, with exception of postmenopausal women aged 50–64 years [6]. In a study using data from the Breast Cancer Surveillance Consortium (BCSC), Kerlikowske et al. found that in an annual screening program, DM screening sensitivity was also significantly lower in the higher BI-RADS breast density categories than in the lower BI-RADS categories for women aged 50–74 years [7]. However, in another paper by Kerlikowske et al., also using BCSC data, no significant differences in screening sensitivity between breast density categories was found, when DM was used [23]. Finally, in a recently published study of Weigel et al., where data of the German biennial screening program was used, screening sensitivity was found to be lower in the higher as compared to the lower breast density categories. In that study, screening sensitivities of 100 and 50% were found for the lowest and the highest density category, respectively [22].

Although the results in the above studies are not completely consistent, the majority of them showed that screening performance is still negatively influenced by breast density when DM is used instead of FSM. This is also found in the current study.

Four out of six above-mentioned studies were conducted in the USA [6, 7, 17, 23]. The only European study determining the influence of breast density on digital mammography screening performance was the recently published study of Weigel et al. [22]. However, our study is the first to determine the effect of automatically assessed volumetric mammographic density on DM screening performance in a European population-based screening setting. There are three notable differences between European and American screening programs: (1) recall rates are below 5–7% in Europe and around 8–10% in the USA [23–29]; (2) double-reading, which is also used in this study, is common in European screening programs, but not in the USA [30]; (3) the screening interval is different. Biennial screening is common in European countries, while in the USA, women are mostly screened yearly [30].

When looking at the interval cancers diagnosed within the first year after a negative screening mammogram, we found that in the lower density categories, only a small part of the interval cancers were found in the first year after a negative screening examination, and most were found in the second year, whereas in women with extremely dense breasts, this was the other way around. Although a one-year screening interval instead of a 2-year screening interval would probably result in a higher program sensitivity in all density groups, this will happen to a larger extent in the women with fatty breasts than in those with extremely dense breasts, resulting in larger differences in screening sensitivity across density categories.

When only invasive cancers instead of both invasive and in situ cancers were taken into account, the screening sensitivity decreased most notably in VDG4. This indicates that the detection of invasive breast cancers in DM screening is hampered to a larger extent than the detection of in situ breast cancers (Table 3). A possible explanation for this is that the visibility of microcalcifications, that often are the hallmark of ductal carcinoma in situ (DCIS) on mammography [20], is not hampered as much in dense tissue as the visibility of invasive breast cancers. 89.4% of the DCIS in our study was accompanied by microcalcifications.

False positive rates were found to be higher in women with dense breasts compared to women with nondense breasts. Similar trends were found in two American studies using BCSC data [7, 31].

When looking at the tumor characteristics of screen-detected breast cancers, we observe a significant linear trend for the proportion of invasive tumors over breast density categories (*p*-trend = 0.03). In addition, the size of screen-detected cancers and the proportion of positive lymph node status among screen-detected cancers seem to be larger in denser breasts. However, no significant linear trend was found for screen-detected tumor size and positive lymph node status proportion across the four density categories (*p*-trend_size_ = 0.10 and *p*-trend_lymph node status_ = 0.08).

It should be noted that the four density categories (VDGs) used in this study are comparable to the 4th edition BI-RADS density categories. Although in 2013 the 5^th^ BI-RADS density edition was introduced, we here still used the VDG categories comparable to the 4th edition, to enable better comparison with previous studies.

A limitation of this study is that during the study period, the MLO view was the standardly acquired view for the subsequent screening rounds and CC views were only taken in addition to MLO during the first screening round or by indication during subsequent rounds. As a result, breast density was determined based on only MLO views for some examinations and on both MLO and CC views for other examinations in our main analysis. Volpara’s PDV measured on CC views tends to be somewhat higher than on MLO views [32]. As CC views are more often performed among women with dense breasts and women with a suspicious region on their MLO view, breast density might be somewhat artificially elevated for these women. Our sensitivity analysis using VDG categories based on PDV from the MLO views only did not lead to different conclusions. Screening sensitivity is presumably higher when both MLO and CC views are available compared to MLO views only. Therefore, standardly taking both MLO and CC views would lead to higher sensitivity, particularly in women with fatty breasts as they are the ones who most often receive MLO views only. This would lead to larger differences in screening performance across breast density categories.

Strengths of this study are the large sample size and the fact that the digital mammograms were acquired in routine screening. In addition, we used a fully automatic method to determine PDV, which was possible because unprocessed image data were archived. In several studies, this automatic method (Volpara) showed to be correlated with BI-RADS breast density and to give comparable breast cancer risk estimations as with BI-RADS breast density [13, 15, 16]. In addition, it has been validated against MRI [14]. Volpara gives objective and reproducible density measurements, representing the amount of dense tissue rather than the size of the dense tissue projection as measured by area-based methods.

In summary, in a large screening population, where DM was used for screening and a fully automatic method (Volpara) was used to determine PDV, breast density was found to significantly hamper the detection of breast tumors. This is shown by a lower screening sensitivity in women with dense compared to those with nondense breasts, which existed despite a higher recall rate for women with dense breasts. These findings are in line with results of most studies using visually assessed BI-RADS density on digital mammograms. Since measuring breast density fully automatically has higher reproducibility than visual assessment, this automatic method could help with facilitating a more tailored screening, such as supplemental screening for women with dense breasts.

## Abbreviations

DM: Digital mammography
ACR: American College of Radiology
USA: United States of America
BI-RADS: Breast imaging-reporting and data system
MRI: Magnetic resonance imaging
CC: Craniocaudal
MLO: Mediolateral oblique
ICD-O: International classification of diseases for oncology
PDV: Percentage dense volume
VDG: Volpara density grade
GEE: Generalized estimating equations
DR: Direct radiography
DMIST: Digital mammographic imaging screening trial
BCSC: Breast cancer surveillance consortium
FSM: Film-screen mammography
PPV: Positive predictive value
CI: Confidence interval
DCIS: Ductal carcinoma in situ
